# Phylogenetic study of *Theileria ovis* and *Theileria lestoquardi* in sheep from Egypt: Molecular evidence and genetic characterization

**DOI:** 10.14202/vetworld.2021.634-639

**Published:** 2021-03-13

**Authors:** Amira Adel Al-Hosary, Ahmed ElSify, Akram A. Salama, Mohamed Nayel, Ahmed Elkhtam, Layla Omran Elmajdoub, Mohamed Abdo Rizk, Manal Mohammed Hawash, Mohammad Ali Al-Wabel, Abdulaziz M. Almuzaini, Laila Salah El-Din Ahmed, Anand Paramasivam, Suresh Mickymaray, Mosaab A. Omar

**Affiliations:** 1 Department of Animal Medicine (Infectious Diseases), Faculty of Veterinary Medicine, Assiut University, Assiut 71526, Egypt; 2 Department of Animal Medicine and Infectious Diseases, Faculty of Veterinary Medicine, University of Sadat City, Sadat City 32897, Menoufia, Egypt; 3 Department of Parasitology, Faculty of Veterinary Medicine, University of Sadat City, Menoufia 32897, Egypt; 4 Department of Zoology, College of Science, Misurata University, Maturate, Libya; 5 Department of Internal Medicine and Infectious Diseases, Faculty of Veterinary Medicine, Mansoura University, Mansoura 35516, Egypt; 6 Department of Public Health, Faculty of Applied Medical Sciences, King Khalid University, Saudi Arabia; 7 Department of Gerontological Nursing, College of Nursing, Alexandria University, Egypt; 8 Department of Veterinary Medicine, College of Agriculture and Veterinary Medicine, Qassim University, 51452 Qassim, Saudi Arabia; 9 Department of Basic Medical Sciences, College of Dentistry, Al-Zulfi, Majmaah University, Majmaah 11952, Riyadh Region, Saudi Arabia; 10 Department of Biology, College of Science, Al-Zulfi, Majmaah University, Majmaah 11952, Riyadh Region, Saudi Arabia; 11 Department of Parasitology, Faculty of Veterinary Medicine, south valley university, Qena 83523, Egypt

**Keywords:** Egypt, polymerase chain reaction, phylogeny, *Theileria lestoquardi*, *Theileria ovis*

## Abstract

**Background and Aim:**

Ovine theileriosis caused by *Theileria ovis* and *Theileria lestoquardi* is an important infectious disease affecting small ruminants in regions of the tropic and subtropic zones. There is limited studies about ovine theileriosis in Egypt; so the present study aims to assess the occurrence of ovine theileriosis in Egypt at the molecular level.

**Materials and Methods:**

Blood samples were collected from 115 randomly selected sheep, which were apparently healthy; the ages of the sampled sheep ranged from 1 to 5 years old, from a local breed (barkae and balade), and showed no symptoms indicating infection with *Theileria* spp. The study was conducted in three governorates representing Lower Egypt (Menoufia and Beheira) and Upper Egypt (El-Wady El-Geded). All blood samples were subjected to polymerase chain reaction (PCR) and semi-nested PCR to target *Theileria* spp. *18S* rRNA genes. Positive samples were sequenced, and these sequences were analyzed using nucleotidebasic local alignment search tool (BLAST).

**Results:**

Six animals (5.22%) were PCR-positive carriers for ovine theileriosis. Nucleotide BLAST and phylogenetic analyses of the six obtained sequences showed that *T. ovis* was present in five animals (4.37%) in Menoufia (n=2) and El-Wady El-Geded (n=3), whereas *T. lestoquardi* was detected in 1 animal (0.87%) in El-Wady El-Geded.

**Conclusion:**

This study is the first to provide molecular evidence, genetic characterization, and phylogenetic analysis of ovine *Theileria* spp. in Egypt. Specifically, *T. lestoquardi* and *T. ovis* carrier statuses of sheep were confirmed. These results highlight the importance of developing an effective control strategy against ovine theileriosis carriers that might develop and/or spread theileriosis.

## Introduction

Ovine theileriosis is one of the most important malignant diseases infecting small ruminants, including sheep, in regions of the tropic and subtropic zones [1]. These infectious diseases are mainly caused by *Theileria ovis* and *Theileria lestoquardi*, which are transmitted by several tick species, including *Hyalomma* spp. and *Haemaphysalis* spp. [2,3]. In particular, *T. lestoquardi* is a widespread pathogen in the tropical and subtropical regions of Africa, the Middle East, East and South Europe, and Asia [4-6].

Mortality rates of up to 73% have been reported for malignant ovine theileriosis [7], with typical symptoms including fever, lethargy, cough, lymphadenopathy, and weight loss [8]. Usually, ovine theileriosis is diagnosed based on observations, symptoms, serological methods, and microscopic examination of Giemsa-stained blood or lymph node smears, by which the parasitic stages, especially piroplasms and schizonts, can be detected [9,10]. However, these diagnostic methods exhibit relatively low sensitivity and specificity [9,10].

In contrast, the use of polymerase chain reaction (PCR) to investigate DNA from parasites is highly specific, is sensitive, enables detection of infectious disease, and allows exploration of the causative agents of the disease [11-13]. In sheep, *Theileria* species have been effectively identified using diverse molecular tools according to the occurrence of *18S* rRNA genes located in the hypervariable V4 region. Existing primers have been used to find *18S* rRNA genes as there is a cross-reaction with the species *Theileria annulata* and *T. lestoquardi* [14,15]. In the previous studies, *T. ovis* was found in sheep in Egypt [16-18]. Although each of these studies identified the infection using ELISA or PCR techniques, the positive samples were not subjected to molecular characterization or phylogenetic analysis. Moreover, there is currently a lack of consistent information about *T. lestoquardi*-mediated infections in sheep.

Therefore, the present study aimed to investigate the occurrence of *T. ovis* and *T. lestoquardi* among apparently healthy sheep in three administrative Governorates of Egypt using molecular techniques, genetic characterization, and phylogenetic analysis.

## Materials and Methods

### Ethical approval

Ethical approval is not needed for this study; however, the animals were treated in rigid adherence to the accepted standards for the humane treatment of animals and according to the guidelines of the Committee of Animal Care and Welfare, Faculty of Veterinary Medicine, University of Sadat City.

### Study area and period

**Figure-1:**
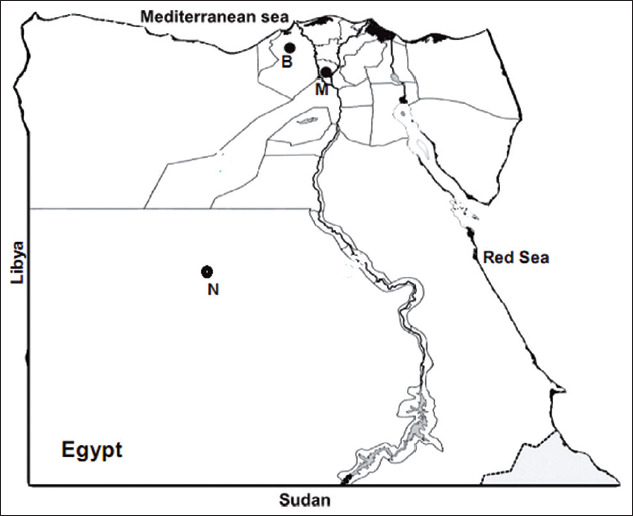
Study area in Egypt. The blood samples were obtained from animals reared in N: New Valley province. M: Menoufia province. B: Beheria province as specified by bullet points [Source: Google.com/maps/search].

The current study included 115 apparently healthy balade and barkae sheep aged (1-5) years that had been reared in two governorates of Lower Egypt (Menoufia and Beheira) and one province of Upper Egypt (EL-Wady EL-Geded) (Figure-1). The sheep were managed by semi-intensive or extensive practices. Samples were collected during June and July 2014.

### Sample collection

Two blood samples were obtained from each individual animal. The first sample, taken for DNA extraction purposes, was collected from the jugular vein. The blood was placed in a tube containing EDTA, labeled, and maintained at –200C. The second sample, collected as thin blood smears for microscopic examination, was obtained from the ear vein. These thin blood smears were normally collected from venous blood, fixed with methanol, and stained using Giemsa stain. Subsequently, the smears were observed under a microscope to identify the occurrence of tick pathogens. Using an oil immersion lens (1000×), approximately 50 microscopic fields were observed for piroplasms of *Theileria*. The occurrence of each piroplasm was measured carefully.

### Extraction and amplification of DNA

DNA was extracted using a QIAamp DNA Mini Kit according to the manufacturer’s instructions (Qiagen, UK). 18S rRNA-encoding genes of both *T. ovis* and *T. lestoquardi* were detected by PCR and semi-nested PCR [19]. The amplification was achieved using the respective forward and reverse primers RLB-F2 (5′-GAC ACA GGG AGG TAG TGA CAA G-3′ and 5′-CTA AGA ATT TCA CCT CTG ACA GT-3′). In addition, the nested forward primer RLB-F (5′-GAC AAG AAA TAA CAA TAC RGG GC-3′) was used. All primers were obtained from Qiagen, UK. The reaction was conducted in an overall volume of 25 mL, comprising master mix (12.5 mL; Promega), forward primer (1 mL), reverse primer (1 mL), DNA (5 mL), and water (5.5 mL). The PCR program used was as follows: 940C for 10 min; three cycles of 20 s at 940C, 30 s at 670C, and 30 s at 720C; after every second cycle, the temperature of the annealing phase was reduced by 20C. Furthermore, 40 cycles of 20 s at 940C, 30 s at 570C, and 30 s at 720C, with a final extension at 720C for 7 min were performed in an automated Thermocycler (Biometra Thermal Cycler, Germany). The expected PCR product lengths for *T. ovis* and *T. lestoquardi* were 460 and 520 bp, respectively. In total, a 10 mL volume of PCR product was used for electrophoresis with 1.5% ethidium bromide (Sigma-Aldrich, U.S.A.) and agarose gel in TBE buffer. Subsequent visualization was performed under a UV transilluminator [20,21]. The PCR amplicons exhibited significant band intensity, which was extracted from the agarose gel using a gel extraction kit (QIAquick, QIAGEN, UK).

### DNA sequencing analysis

The bands for the respective positive samples were then cloned with a plasmid vector using a TOPO TA Cloning Kit with PCR 2.1 (Invitrogen, USA). Using an ABI PRISM 3100 Genetic Analyzer with an Applied Biosystems sequencer (Molecular Biology Unite, Assiut University, USA), two clones were immediately sequenced for individual amplicons. The obtained sequences were amended and ranged using MEGA (version 7.0.7) (https://www.megasoftware.net) before being analyzed with the basic local alignment search tool (BLAST) algorithms from the National Center for Biotechnology Information using available databases. Phylogenetic analysis was performed using the neighbor-joining method and the outcomes of sequences and homologous sequences from various other regions of the world [22].

## Results

All thin blood smears were negative for the occurrence of any piroplasm stage inside the blood cells. Data from the PCR assays targeting the *18S* rRNA gene showed that positive samples were found in 6 of 115 sheep (5.21%). Of these six sheep, two were from the Menoufia Governorate (8% of 25 sheep) and four were from the El-Wady El-Geded Governorate (6.34% of 63 sheep). Positive samples were not detected in the Behira Governorate (from 27 sheep). Based on nucleotide BLAST analysis of the six sequences, five were *T. ovis* (two in Menoufia Governorate and three in El-Wady El-Geded Governorate), and one was *T. lestoquardi* (from El-Wady El-Geded Governorate).

The two *T. ovis* sequences isolated from sheep in the Menoufia Governorate (AB986193 and AB986194) had the same length (434 bp) and had 99.8% identities when compared. In contrast, the three *T. ovis* sequences isolated from sheep in El-Wady El-Geded (KY494648, KY494649, and KY494650) were 395, 395, and 391 bp long, respectively, with 97.0-98.7% identities. Among *T. ovis* isolates from the Menoufia Governorate and El-Wady El-Geded Governorate, sequence identities were 89.6-91.0%.

**Figure-2:**
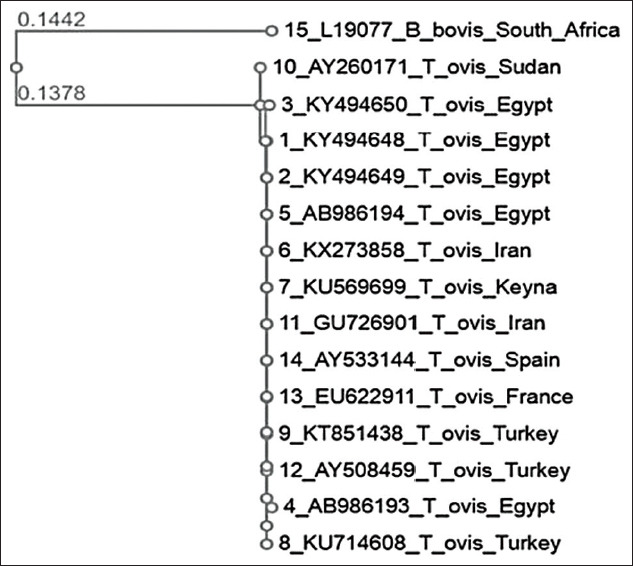
The phylogenetic tree analysis of *Theileria ovis* using the 18S rRNA gene sequence. This tree was drawn by BLAST pairwise alignments. The scale bar signifies 0.01 variations per nucleotide. Numbers at nodes signify proportions of clades in 1000 bootstrap replications of information.

**Figure-3:**
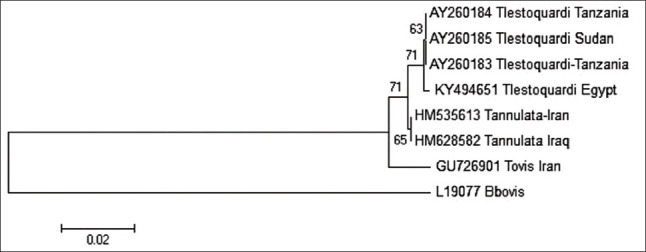
The phylogenetic tree analysis of *Theileria lestoquardi* using the 18S rRNA gene sequence. This tree was drawn by basic local alignment search tool pairwise alignments. The scale bar signifies 0.02 variations per nucleotide. Numbers at nodes signify proportions of clades in 1000 bootstrap replications of information.

As shown in Figure-2, *T. ovis* sequences from the present study included two haplotypes based on a neighbor-joining tree for the *18S* rRNA gene. They were grouped in the same clade as *T. ovis* sequences obtained from various regions, including Asia, Africa, and Europe. The only *T. lestoquardi* sequence obtained in the present study (KY494651), which was from an animal reared in the El-Wady El-Geded Governorate, was 416 bp long. As shown in Figure-3, this sequence exhibited 99.5% identity with *T. lestoquardi* sequences AY260183 and AY260184 isolated from small ruminants in Tanzania and the AY260185 sequence isolated from sheep in Sudan. Moreover, it showed 100% identity with KF771185 isolated from a buffalo in Assiut Governorate in Upper Egypt.

## Discussion

*Theileria* parasites are distributed globally and cause severe tick-borne diseases in animals, inflicting a substantial economic burden on rural and agricultural societies [23]. Theileriosis in small ruminants is caused by several species and new uncharacterized genotypes including *T. lestoquardi* (formerly *T. hirci*), *T. ovis*, *T. annulata*, *Theileria* *recondita*, *Theileria* *uilenbergi*, *Theileria* *separata*, *Theileria* *luwenshuni*, *Theileria* sp. OT1, *Theileria* sp. OT3, and *Theileria* sp. MK [4,23-29]. *Theileria ovis* and *T. separata* are known for their lack of pathogenicity [30], whereas *T. lestoquardi* is classified as a malignant parasite due to the high mortality rates resulting from the infections it causes [31]. In the present study, blood smears and microscopic observations of Giemsa-stained samples from 115 healthy sheep were negative for ovine theileriosis, and detecting low levels of parasitemia were not possible [32]; therefore, a PCR assay and sequencing analysis targeting the *18S* rRNA gene were used to obtain positive bands that were further used to diagnose ovine theileriosis.

Of 115 DNA samples, 6 samples (5.21%) were positive for ovine theileriosis. Nucleotide BLAST analysis revealed that five sequences were *T. ovis* (two from Menoufia Governorate with accession numbers AB986193 and AB986194; three from El-Wady El-Geded Governorate with accession numbers KY494648, KY494649, and KY494650). Only one of the generated sequences was *T. lestoquardi* (from El-Wady El-Geded Governorate with accession number KY494651). The higher detection rate of *T. ovis* over *T. lestoquardi* follows the previously detected rates [33,34]. Similarly, both *T. lestoquardi* and *T. ovis* have previously been shown to be primary infectious vectors of ovine theileriosis in Egypt through PCR [17,35].

*T. lestoquardi* was detected only in El-Wady El-Geded Governorate, suggesting that its prevalence is affected by geographical and climatic factors [33,36,37]. *T. ovis* sequences obtained in the present investigation showed highly similar identities (99-100%) to *T. ovis* sequence from Iran (KX273858) [38], and they were grouped in the same clade as sequences of *T. ovis* from the various regions of Asia, Africa, and Europe [38-40]. Moreover, these sequences showed strong similarities with corresponding sequences from sheep in the same clade from the Mediterranean area including Turkey, France, Spain, and Iran [25,39,41].

Only one *T. lestoquardi* sequence (KY494651) was obtained in this study. It originated from an animal reared in the El-Wady El-Geded Governorate and exhibited 99.5% identity with *T. lestoquardi* sequences AY260183 and AY260184 isolated from small ruminants in Tanzania and the AY260185 sequence isolated from sheep in Sudan, as well as 100% identity with KF771185 isolated from a buffalo in Egypt [42]. These high sequence identities for the *18S* rRNA gene among *T. lestoquardi* sequences indicate that *T. lestoquardi* is transmitted between large and small ruminants, as previously suggested [43].

This is the first study to address the molecular characterization of *T. ovis* and *T. lestoquardi* in Egypt based on sequencing; however, the study focused only on sheep. In addition, the sample size used in this investigation was low, which means that the results are not conclusive. Hence, further studies are required in which larger sample sizes, other small ruminants, and more epidemiological data are considered. Such studies will help conclusively evaluate the risk factors connected to *T. ovis* and *T. lestoquardi* infection in Egypt.

## Conclusion

This study provides molecular evidence and genetic characterization for *T. ovis* and *T. lestoquardi* infection in sheep from Egypt based on sequencing. The results confirm the carrier status of *T. lestoquardi* and *T. ovis*, which could lead to the development and/or spread of theileriosis. Thus, the findings suggest the importance of developing effective control strategies, such as immunization and vector control, against ovine theileriosis in Egypt.

## Authors’ Contributions

AE1, AAA, and MAO: Conceived and scheduled the tests. AE1, AAA, AAS, MN, and MAO: Piloted the experiments. MAR, MMH, AE2, LSEA, and MN: Examined the data. MAR, AMA, MMH, MAAW, AMA, AP, and MS: Delivered reagents/materials/analysis tools. LOE, MAO, AE, AAA, AP, and SM: Wrote and critically revised the manuscript. All authors read and approved the final manuscript.
